# NLRP7, Involved in Hydatidiform Molar Pregnancy (HYDM1), Interacts with the Transcriptional Repressor ZBTB16

**DOI:** 10.1371/journal.pone.0130416

**Published:** 2015-06-29

**Authors:** Heike Singer, Arijit Biswas, Nicole Nuesgen, Johannes Oldenburg, Osman El-Maarri

**Affiliations:** Institute of Experimental Hematology and Transfusion Medicine, University of Bonn, Bonn, Germany; VU University Medical Center, NETHERLANDS

## Abstract

Mutations in the maternal effect gene *NLRP7* cause biparental hydatidiform mole (HYDM1). HYDM1 is characterized by abnormal growth of placenta and lack of proper embryonic development. The molar tissues are characterized by abnormal methylation patterns at differentially methylated regions (DMRs) of imprinted genes. It is not known whether this occurs before or after fertilization, but the high specificity of this defect to the maternal allele indicates a possible maternal germ line-specific effect. To better understand the unknown molecular mechanism leading to HYDM1, we performed a yeast two-hybrid screen against an ovarian library using NLRP7 as bait. We identified the transcriptional repressor ZBTB16 as an interacting protein of NLRP7 and verified this interaction in mammalian cells by immunoprecipitation and confocal microscopy. Native protein analysis detected NLRP7 and ZBTB16 in a 480kD protein complex and both proteins co-localize in the cytoplasm in juxtanuclear aggregates. HYDM1-causing mutations in NLRP7 did not show altered patterns of interaction with ZBTB16. Hence, the biological significance of the NLRP7-ZBTB16 interaction remains to be revealed. However, a clear effect of harvesting ZBTB16 to the cytoplasm when the NLRP7 protein is overexpressed may be linked to the pathology of the molar pregnancy disease.

## Introduction

Hydatidiform mole (HYDM1, MIM #609661) is a human abnormal pregnancy with excessive proliferation of the trophoblast, degeneration of chorionic villi and abnormal embryonic development. It is usually the consequence of an androgenetic contribution. While the event of an androgenetic mole is sporadic and not recurrent, in rare familial cases, a recurrent form of the disease occurs that is characterized by a biparental genetic contribution. Genetic mapping identified *NLRP7* located on chromosome 19q13.42 as the maternal effect gene responsible for HYDM1 [1]. To date, approximately 36 HYDM1 families have been reported with various mutations in *NLRP7* affecting both alleles in the mother, either homozygous or compound heterozygous, leading to recurrent pregnancy loss [2]. Furthermore, patients with only one molar pregnancy and at least three spontaneous abortions are affected by non-synonymous variants (NSV) in NLRP7, all heterozygous missense variants, suggesting a milder phenotype associated with the disease.

NLRP7 is a protein belonging to one of the four major families of pattern recognition receptors called nucleotide-binding oligomerization domain (Nod)-like receptors (NLRs) that are also known to act as defenders against microbes and chemicals in the innate immune system. NLRP7 is a member of the PYD domain-containing NLRP group, which allows binding and activation of caspase-1 in an inflammasome resulting in subsequent maturation and secretion of IL-1ß and IL-18 in human macrophages [3]. The protein comprises four functional domains, the N-terminal effector domain PYRIN (PYD), a central NACHT domain for initiating oligomerization by binding ATP on the conserved sequence motif WalkerA/Ploop, a NACHT-associated domain (NAD) and a C-terminal leucin rich repeat region for sensing PAMPs and DAMPs. Recently, we identified the NAD domain as likely physical mediator for the oligomeric assembly. An Apaf1-based *in silico* model and an extensive interdomain interaction screen of NLRP7 showed that the NAD is buried inside the LRR domain during the inactive state of NLRP7, while upon activation/oligomerization, it exposes from any sequestration and interacts with the NACHT domain of a second NLRP7 molecule [4]. Apart from their inflammatory role, NLRP2 and NLRP5 are also suggested to play a role in mammalian reproduction [5, 6]. A homozygous mutation in *NLRP2* was found to be responsible for the imprinting disorder Beckwith-Wiedemann syndrome (BWS) in two offspring of the affected mother [7]. Expression analysis of different NLRP genes revealed specific expression in human gametes and embryos. NLRP2, 4, 5, 7, 8, 9, 11, 12, 13 and 14 are highly expressed in MI and MII oocytes, whereas this expression sharply decreases in a two and three-day old developing embryo [8]; only NLRP2 and NLRP7 are again showing a sharp increase of expression in the developing embryo on day 5.

Several DNA methylation analysis studies on molar tissues, performed by our group and others, revealed a general trend of decreased methylation levels at maternally methylated DMRs (paternally expressed genes), such as SNRPN, PEG3 and KCNQ1OT1, indicating a possible fault in the establishment of maternal methylation marks during oocyte growth (i.e. on the future maternal allele) [9, 10]. In addition, others and we found a gain of methylation levels at the paternally methylated DMR NESP55, on the maternal allele [9–11]. In addition, our study also detected a gain of methylation at the paternally methylated primary DMR H19 [9].

To date, there is no evidence on how mutations in *NLRP7* lead to the abnormal patterns of DNA methylation at the imprinted loci on the maternal allele. Yet, the fact that multiple imprinting control centers (ICs) are affected suggests an imprinting regulatory mechanism that may work in *trans* [12]. However, NLRP7 mutation in one life birth demonstrated abnormal methylation patterns beyond imprinted genes, albeit with a ratio that was highly biased to imprinted loci and only a relatively small number of affected non-imprinted CpG sites [13, 14].

In addition to NLRP7, Parry et al. identified mutations in *KHDC3L* of patients suffering from the same HYDM-specific phenotype termed HYDM2 (MIM #614293) [15]. *KHDC3L* is located within a reproduction related gene cluster on chromosome 6 [16], it has a similar temporal expression pattern in oocytes like *NLRP7* and shows co-localization with NLRP7 [15, 17]. It is believed that both proteins might participate in the same complex/pathway during human oogenesis or embryogenesis.

In order to gain more insights into the intracellular function of NLRP7, we used a yeast two-hybrid system to screen a human ovarian cDNA library for novel NLRP7 interacting partners. We identified ZBTB16 (PLZF), a well-known transcriptional repressor protein, as a potential interacting partner of NLRP7. ZBTB16 is a member of the Krüppel-like zinc finger protein family and is involved in major developmental and biological processes, such as spermatogenesis, hind limb formation, hematopoiesis and immune regulation [18]. The protein interaction was further verified by immunoprecipitation and confocal microscopy in mammalian cells. Deletion mapping studies identified multiple interaction sites between ZBTB16 and NLRP7. Blue Native PAGE analysis showed that both proteins are present at a 480 kD protein complex and that co-expression of NLRP7 and ZBTB16 might lead to an active nuclear export of ZBTB16 or impede its nuclear import, so that both proteins co-localize in cytoplasmic juxtanuclear aggregates.

## Materials and Methods

### Cloning DNA constructs

**Fig 1:**
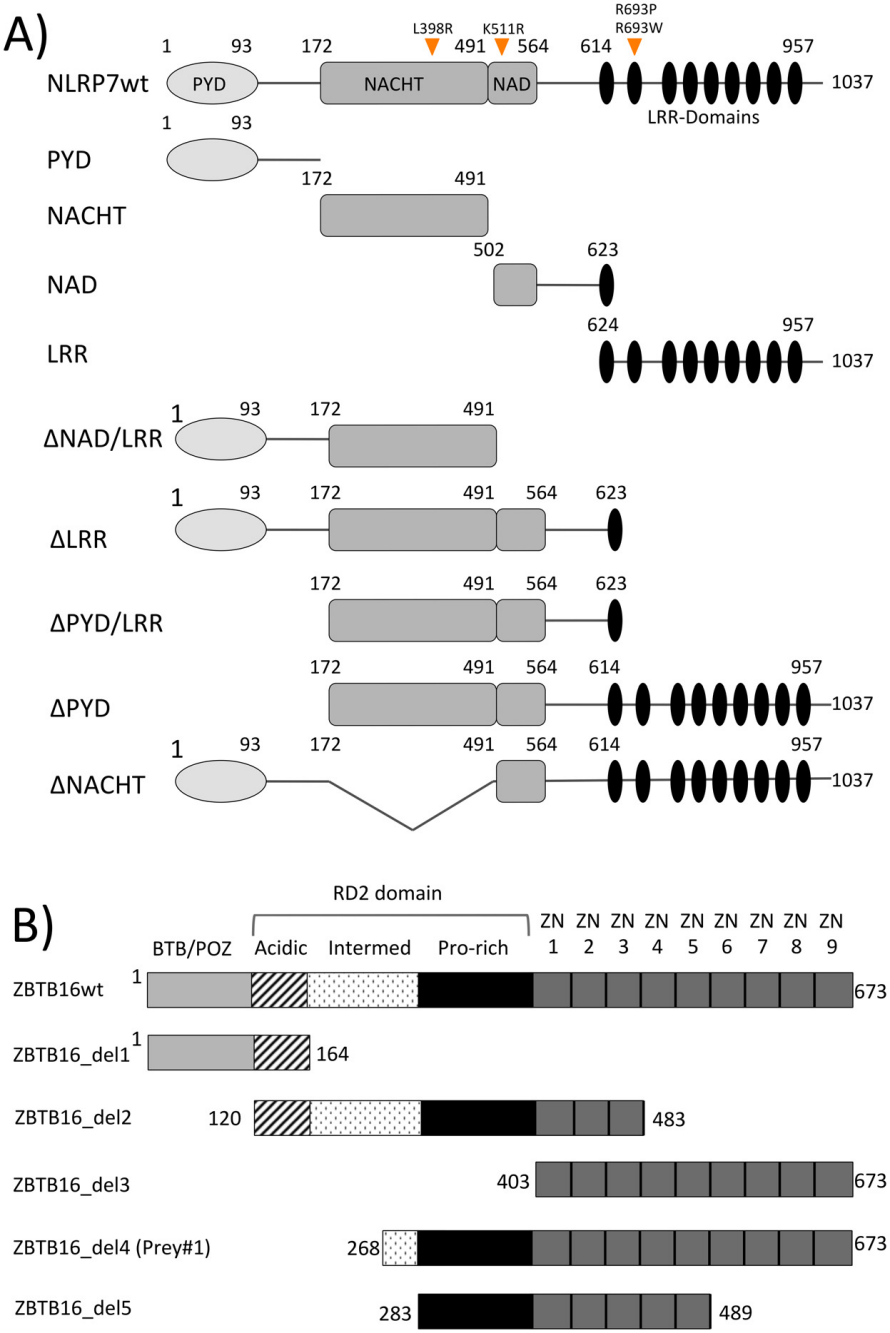
Domain structure of NLRP7 and ZBTB16. **A.** Domain structure of NLRP7. Location of analyzed HYDM1-causing mutations L398R, R693P, R693W and non-synonymous variant (NSV) K511R are marked with orange arrowheads. Apart from a full-length construct and the four individual NLRP7 domains, five additional deletion constructs were generated, either lacking one or two domains at a time. **B.** Domain structure of the transcription factor ZBTB16 identified as interaction partner by the yeast two-hybrid screening. Apart from the full-length construct, containing an N-terminal BTB/POZ, a RD2 and nine zinc-fingers, five additional ZBTB16 deletion constructs were generated (ZBTB16_del1-5). ZBTB16_del4 (aa 268–673) corresponds to “prey#1” identified by the yeast two-hybrid screen.

The full-length cDNA for human NLRP7, ZBTB16 (pCMV-SPORT6 IMAGE ID 4944546; Imagenes), KHDC3L (pCR4-TOPO CloneID 40146866; ThermoScientific), the NLRP7 individual domains PYD, NACHT, NAD, LRR and the NLRP7 deletion constructs ΔNAD/LRR, ΔLRR, ΔPYD/LRR, ΔPYD, ΔNACHT (Fig 1A and 1B) were cloned by the Gateway system (Invitrogen) into GAL4 yeast expression vectors pGBKT7/pGADT7 (Clontech) and by restriction-free cloning into Flag- or Myc-containing pcDNA3.1 vector (Invitrogen) as previously reported [4]. Additionally, ZBTB16 deletion constructs del1 (aa 1–164), del2 (aa 403–673), del3 (aa 268–673 = Y2H_Prey#1), del4 (aa 283–489) and del5 (aa 120–483) were cloned into Myc-containing pcDNA3.1. All constructs used for confocal microscopy were cloned into pEGFP-C1 and pDsRed2-N1 (Clontech). The integrity of all clones was confirmed by direct sequencing of the whole insert.

*NLRP7* missense mutations L398R, R693P and R693W are hotspot mutations. In addition, we chose the non-synonymous variant K511R as the appearance of NSVs in NLRP7 results in a diagnostic overlap between patients with a single molar pregnancy or 2–3 spontaneous abortions. Each mutation/NSV was introduced into the full-length construct by site-directed mutagenesis as previously reported [4]. All primers used are listed in S2 Table.

### Yeast two-hybrid

A LexA-Dir based yeast two-hybrid system was used to screen NLRP7 against a human ovarian cDNA library (DUALhybrid screening, Dualsystems; cDNA library from Clontech Cat#638822; library is constructed from five Caucasians donors aged between 30 and 60 yrs. whose cause of death was indicated as trauma). For further verification,human cDNA of ZBTB16 was fused to GAL4 DNA-BD (pGBKT7-Vector) and screened against Gal4 DNA-AD (pGADT7-Vector) NLRP7 fusion constructs with the MATCHMAKER GAL4 two-hybrid system (Clontech) as previously reported [4].

### Co-immunoprecipitation

Cell culture, transfection, immunoprecipitation (IP) and immunoblotting (IB) of HEK293T cells (ATCC) and cell lysates were done as previously described [4]. Neither NAD nor PYD single domains could be included for IP/IB as they produced lower expression and caused difficulties in the detection after western blots. The quantitative evaluation of the immunoprecipitated products was calculated by determining the band density on the western blots using the FluorChemSP AlphaEaseFC software version 6.0.0 (Alpha Innotech); to correct for the differences in expression between different NLRP7 constructs and the ZBTB16 input between different reactions, in each lane, we applied the following formula:

[Co−IP ZBTB16 / ratio (IP−NLRP7 domains / IP−NLRP7 full−length)] / input ZBTB16 x 100.

### Blue Native PAGE

Blue Native gel electrophoresis was performed using the Bis-Tris NativePAGE system from Invitrogen according to the manufacturer´s protocol. HEK293T cells were co-transfected with 2 μg of a Myc- and Flag-tagged expression plasmid. After 48 h, cells were washed twice with PBS (PAA), trypsinized, resuspended in fresh media and centrifuged at 1200g for 2 min. Cells were washed twice with PBS, followed by re-suspension in different concentrations of digitonin (1%-2.5%) in NativePAGE sample buffer (Invitrogen) with protease inhibitor cocktail (Roche). After extensive re-suspension, cell debris was pelleted by ultracentrifugation (100 000g) for 45 min at 4°C. Lysates were separated by NativePAGE using the Novex BisTris gel system according to the manufacturer´s protocol (Invitrogen). After western blotting with NuPAGE transfer buffer containing 0.1% NuPAGE antioxidant and 10% methanol using the XCell II blot module (all from Invitrogen), membrane was blocked over night in milk (5% skim milk powder in 1 x TBS with 5% Tween 20) and washed for 10 min with stripping buffer (15 g glycine, 1 g SDS, 10 ml Tween in 1L aqua dist., pH 2.2) to get rid of residual Coomassie G250. Immunoblotting for Myc- and Flag-tagged constructs was carried out with the same antibodies used after immunoprecipitation.

Native gels were subjected to a second dimension of electrophoresis using SDS-PAGE. A sample separated by a native gel was tailored in a 5 cm slice and placed and incubated in a 15ml tube with the appropriate equilibration buffer system from Invitrogen: reducing solution (4.5 ml LDS sample buffer + 0.5 ml sample reducing agent), alkylating solution (5 ml LDS sample buffer + 28 μl DMA [dimethylacrylamide, Sigma]), quenching solution (4 ml LDS sample buffer + 1 ml ethanol + 0.05 ml sample reducing agent). The equilibrated gel strip was loaded into the well of a precast 2D 10% SDS-PAGE gel (Invitrogen) and complexes were separated at 200V for 30 min. After conventional western blotting, immunodetection of Myc- and Flag-tagged constructs was carried out with the same antibodies used after immunoprecipitation.

### Confocal microscopy

Preparation of HEK293T cells for confocal analysis was done as previously described [4] and examined with an Olympus Fluoview FV 1000 confocal microscope (Olympus).

### Methylation analysis

DNA of transiently transfected HEK293T cells was isolated using the DNeasy Blood&Tissue kit (Qiagen). Bisulfite treatment of DNA was done using the EpiTect kit (Qiagen). PCRs of two paternally methylated (*H19* and *NESP55*) and two maternally methylated (*PEG3* and *SNRPN*) imprinted regions were performed using the listed BiHot primer (S2 Table) [9] and hot-start Taq polymerase HOT FIREPol (Solis BioDyne). For quantitative methylation analysis we used the SIRPH protocol [19].

### Modeling of ZBTB16 and docking on NLRP7

The ZBTB16 protein was modeled on the ITASSER server (http://zhanglab.ccmb.med.umich.edu/I-TASSER/; accessed on 3^rd^ September 2013) using default settings [20]. The individual domains were also remodeled separately on the ITASSER server, again using default settings, and were individually replaced onto the full-length protein model. The final model was subjected to rounds of energy minimization and refinement simulation (to generate the final structure). A threading-based model of the activated NLRP7 (based on the apoptosomal structure of Apaf-1) that we reported on in an earlier publication [4] was used to perform blind docking with the ZBTB16 model on the Cluspro web server http://cluspro.bu.edu/ [21]. The model with the best ranking was chosen on the basis of cluster density.

## Results

### Identification of ZBTB16 as a new NLRP7 binding protein

To identify potential NLRP7 interacting proteins, a human ovarian cDNA library and a human normalized cDNA library were screened using full-length NLRP7 cDNA as bait. Only the screen with an ovarian cDNA library indicated positive interactions (S1 Fig). Ten positive transformants were isolated and a homology search in GenBank using BLAST revealed that prey sequences matched with the C-terminal segment of human ZBTB16 protein overlapping the zinc finger domains and parts of the Prolin-rich region in the RD2 domain. All ten clones were tested for ß-galactosidase activity and growth on SDhigh (SD-ade). Seven out of ten initially positive clones showed ß-galactosidase activity and three out of ten clones showed strong growth on SDhigh. The clone (Prey#1: 268–673) showing activation in both tests was selected for further analysis. Since the ZN-finger domain of ZBTB16 has a specific DNA binding-site, which is also known to bind the LexA consensus binding sequence [22], all further verification analyses were performed in the Gal4 system.

**Fig 2:**
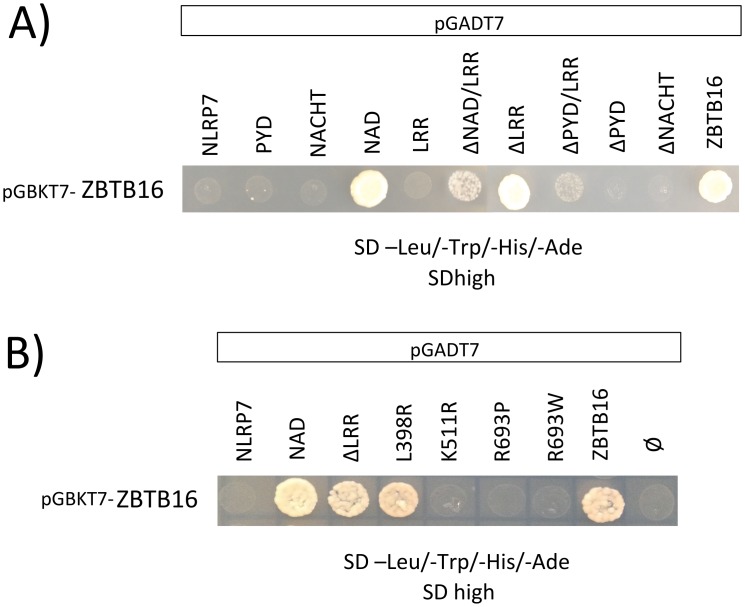
Yeast two-hybrid screen of full-length ZBTB16 against different NLRP7 deletion constructs and against full-length NLRP7 containing different HYDM1-causing mutations. **A.** Yeast two-hybrid screen against the different NLRP7 constructs (fused to the GAL4 activation domain; pGADT7) using full-length ZBTB16 as bait (fused to the GAL4 binding domain; pGBKT7) performed with the Gal4-System (Clontech). ZBTB16 interacted with the highly reactive NAD domain and the LRR deleted constructs. An interaction of ZBTB16 with full-length NLRP7 failed. The self-interaction between full-length ZBTB16 served as positive control as it is already known that ZBTB16 forms a dimer by its N-terminal POZ/BTB domain [30]. **B.** Yeast two-hybrid mutation screen using ZBTB16 as bait (pGBKT7) against full-length NLRP7 containing one of the three HYDM1-causing mutations L398R (NACHT), R693P (LRR), R693W (LRR) or the NSV K511R (all pGADT7). The NACHT domain-associated mutation L398R resulted in the same strong interaction between full-length NLRP7 and full-length ZBTB16 as seen between NAD:ZBTB16 and ΔLRR:ZBTB16.

To further map the exact binding region on both proteins (between ZBTB16 and NLRP7), the cDNA of full-length ZBTB16 was fused to the GAL4 DNA-binding domain and co-transfected as bait with full-length NLRP7, the individual domains of NLRP7 (PYD, NACHT, NAD and LRR) or with one of five NLRP7 deletion constructs (ΔNAD/LRR, ΔLRR, ΔPYD/LRR, ΔPYD and ΔNACHT) into the yeast strain AH109 (Fig 2A, upper row).

Unexpectedly, the full-length ZBTB16 and the full-length NLRP7 did not interact and showed no growth on SDhigh (Fig 2A). However, since our previous study on NLRP7 inter-domain interactions had demonstrated that the presence of LRR has a protective function on the actively interacting part of the protein and thus inhibits the interaction with the full-length self-protein, the same mechanism also explains the lack of interaction of the full-length NLRP7 with ZBTB16 [4]. Despite the above-mentioned negative interaction, positive interactions with ZBTB16 included the NAD domain in NLRP7 and all LRR-deleted NLRP7 constructs that showed interactions with ZBTB16 in variable strength. The deletion constructs ΔNAD/LRR and ΔPYD/LRR interacted weakly with ZBTB16. The latter is an indication of multiple interaction sites, suggesting that the NAD domain is not the sole interacting domain with ZBTB16. Previous data from us on the inter-domain interactions of NLRP7 have shown that the highly reactive and hydrophobic NAD domain is responsible for the oligomeric assembly of the protein [4]. Still, the additional interactions of ZBTB16 with ΔNAD/LRR and ΔPYD/LRR on the high stringent medium suggest the NACHT domain from NLRP7 as additional potential interaction site with ZBTB16, although no interaction between the ZBTB16 and the single NACHT domain of NLRP7 was detected in the yeast system. This is possibly due to the presence of bulky activating domain in the construct interfering with the ability of the lone NACHT domain to interact. Additionally, the correct protein configuration for a positive interaction necessitates the presence of multiple domains to insure the optimum three-dimensional orientation for a given protein-protein interaction.

### The NACHT domain-associated mutation L398R leads to positive interaction between full-length ZBTB16 and full-length NLRP7

Next, we wanted to investigate whether mutations causing HYDM1 disease would alter the interaction with ZBTB16 in the yeast. By investigating the interaction between ZBTB16 and three selected HYDM1-linked mutations L398R, R693P, R693W and the NSV K511R, only the NACHT domain-associated mutation L398R showed a strong gain of interaction in the yeast analysis. All other mutated constructs failed to show any changes in their interaction profile with ZBTB16 and remained negative (Fig 2B). Previous data on *in silico* modeling for the mutation at L398R have shown a shift to a highly hydrophilic and charged arginine residue that most likely induces strong conformational changes in the NLRP7 molecule, exposing potential interaction sites which are normally inaccessible in the fully inactive NLRP7 (in yeast) [4].

### Mapping the interacting domains between NLRP7 and ZBTB16 reveals NACHT-NAD (on NLRP7) and BTB/POZ-ZN7-9 (on ZBTB16) domains as main mediators of the interaction

#### ZBTB16 interaction with different domains of NLRP7

**Fig 3:**
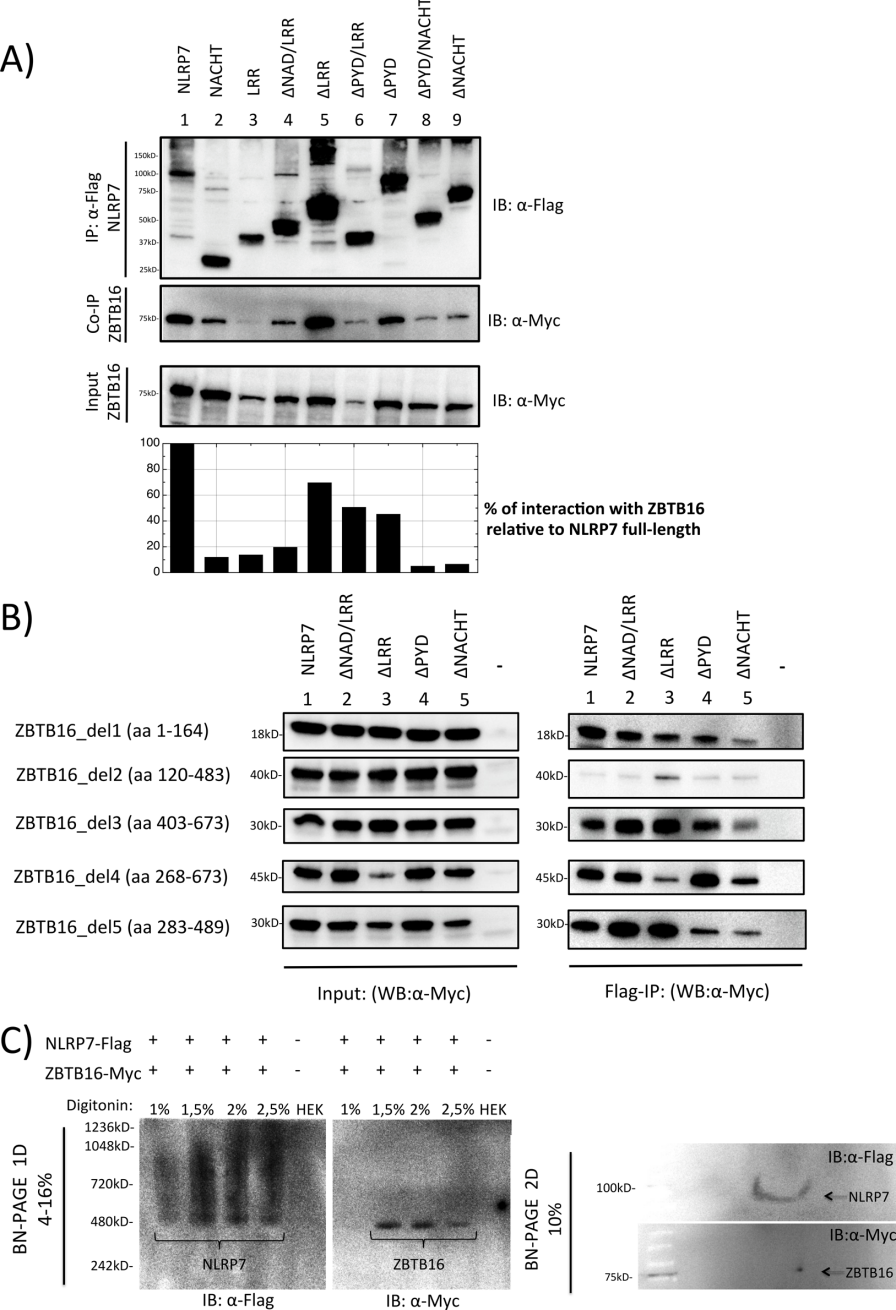
Co-Immunoprecipitation and Blue Native PAGE analysis between NLRP7 and ZBTB16. **A.** Co-immunoprecipitation of transiently transfected ZBTB16 (Myc-tagged) and NLRP7 (Flag-tagged) in HEK293T cells. Immunoprecipitation was done using an anti-Flag specific antibody. Quantification of interaction between NLRP7 and ZBTB16 was determined relative to NLRP7 full-length. **B.** Co-immunoprecipitation of five ZBTB16 deletion constructs del1-5 (Myc-tagged) by immunoprecipitation of full-length NLRP7 (Lane 1) or one of four NLRP7 deletion constructs (lane 2–5; Flag-tagged) using an anti-Flag specific antibody. **C.** Blue Native gel electrophoresis of NLRP7 (Flag-tagged) and ZBTB16 (Myc-tagged) after transient transfection in HEK293T cells. Native protein extraction was performed using different concentrations of digitonin that is indicated above the picture. **Upper panels:** In the first dimension, the native Flag-NLRP7 appears as a smeary oligomer in a broad shift from 480–1000 kD (left side), while native ZBTB16-Myc was detected as a sharp band at 480 kD (right side). **Lower panels:** In the second dimension, the monomeric Flag-NLRP7 is visible as a thin line at 113kD, while ZBTB16-Myc occurs as individual spot at its expected size of 75 kD.

Interaction of complete ZBTB16 (Myc-tagged) with different constructs containing different domain combinations (NACHT, LRR, ΔNAD/LRR, ΔLRR, ΔPYD/LRR, ΔPYD, ΔPYD/NACHT, ΔNACHT) of NLRP7 (Flag-tagged) was done using immunoprecipitation (Fig 3A). By quantifying the bands after western blot analysis we could empirically classify the interaction of ZBTB16 with the different constructs into three different levels. Negative interaction was observed with ΔPYD/NACHT and ΔNACHT (Fig 3A, lanes 8 and 9), slight interaction was observed with NACHT, LRR and ΔNAD/LRR (Fig 3A, lanes 2, 3 and 4), while strong positive interaction was observed with complete NLRP7 (presumably caused by the presence of an activated NLRP7 molecule in HEK293 cells), ΔLRR, ΔPYD/LRR and ΔPYD (Fig 3A, lanes 1, 5 and 7; S2 Fig). This result suggests that absence of the NACHT domain results in negative interactions, while its presence results in intermediate interactions, whereas the additional presence of NAD domain (in addition to the NACHT domain) results in a strong interaction. These immunoprecipitation findings further confirmed the yeast observation results that show that presence of multiple domains in NLRP7 is necessary to guarantee an optimum configuration of the molecule to be able to interact with the ZBTB16. Additionally, none of the single NLRP7 domains can provide strong positive interaction with ZBTB16.

From the above results, we were able to observe one discrepancy in the interaction between the yeast and the mammalian IP experiments, where ΔPYD showed negative interaction in the former but strong interaction in the latter experiment. While we cannot experimentally support this discrepancy, it is likely that absence of the PYD domain in yeast had created a non-favored interaction of NLRP7 with the yeast two-hybrid component (the activator or binding domains) that inhibits an otherwise positive interaction.

#### NLRP7 interaction with different domains of ZBTB16

Knowing that multiple domains are important for interaction on the NLRP7 site with ZBTB16, we next determined the interacting domains on the ZBTB16 molecule. For this purpose, immunoprecipitation was performed with different domains of ZBTB16; this included del1: BTB/POZ-Acidic, del2: RD2-3ZN, del3: 9ZN, del4: Part of Intermed-Pro-Rich-9ZN and del5: Pro-rich-5ZN (Figs 1B and 3B, S5 and S6 Figs). Co-IP of ZBTB16 deletion constructs with complete NLRP7 or with NLRP7 domains revealed a prominent lack of interaction with the ZBTB16 construct (del2) only containing the central RD2 domain and the first three ZN domains (Fig 3B row 2, lanes 1–5). Therefore, here, the data also suggest multiple sites of interactions, namely at the N-terminal (BTB/POZ) and the C-terminal (ZN domain) protein part. Moreover, the weak interaction between the NACHT domain-deleted NLRP7 molecule with all four ZBTB16 deletion constructs re-enforces the stabilizing function of the NACHT domain by providing optimum configuration to NLRP7 (Fig 3B rows 1,3,4 and 5, lane 5).

Co-immunoprecipitations of ZBTB16 with a mutated full-length NLRP7 construct, each containing one of the three HYDM1-causing missense mutations L398R, R693P, R693W and the NSV K511R, did not reveal the clear effect found with the NACHT-domain-located mutation L398R detected in the yeast system (Fig 2B). ZBTB16 always co-precipitated with NLRP7, regardless of any missense mutation present in NLRP7 (data not shown).

### NLRP7 and ZBTB16 are present in a 480kD high molecular weight complex

It is known that inflammasome-forming proteins assemble into high molecular mass multiprotein complexes. While this was already tested for NLRP7 [3], we wanted to examine whether ZBTB16 could be present in the same multiprotein complex (MPC) as NLRP7. To visualize protein assembly, HEK293T cells were co-transfected with Flag-NLRP7 and ZBTB16-Myc and resolved by Blue Native (BN) polyacrylamide gel electrophoresis (PAGE). Immunoblotting was carried out using the two tag-specific antibodies anti-Flag and anti-Myc. Flag-NLRP7 appeared as smeary oligomer in a broad shift from 480–1000 kD, which agrees with the nature of oligomeric protein (Fig 3C; upper panel, left), while ZBTB16-Myc was detected as a sharp band at 480 kD (Fig 3C; upper panel, right).

Subsequently, the subjected samples from the first dimension were transferred and separated to a second dimension by SDS-PAGE. Proteins that represent subunits of the same MPC can be found in one vertical line in the second dimension. Detection of Flag-NLRP7 revealed as a thin line at 113 kD, according to its oligomerized form in the first dimension, while ZBTB16-Myc occurred as an individual spot at 75 kD in the same vertical line of the NLRP7 protein, suggesting co-occurrence in the same complex (Fig 3C lower panels; S7 Fig). These results confirm the picture from the first dimension and indicate that both proteins are present in the same MPC.

### NLRP7 and ZBTB16 co-localize in a cytoplasmic juxtanuclear aggregate

**Fig 4:**
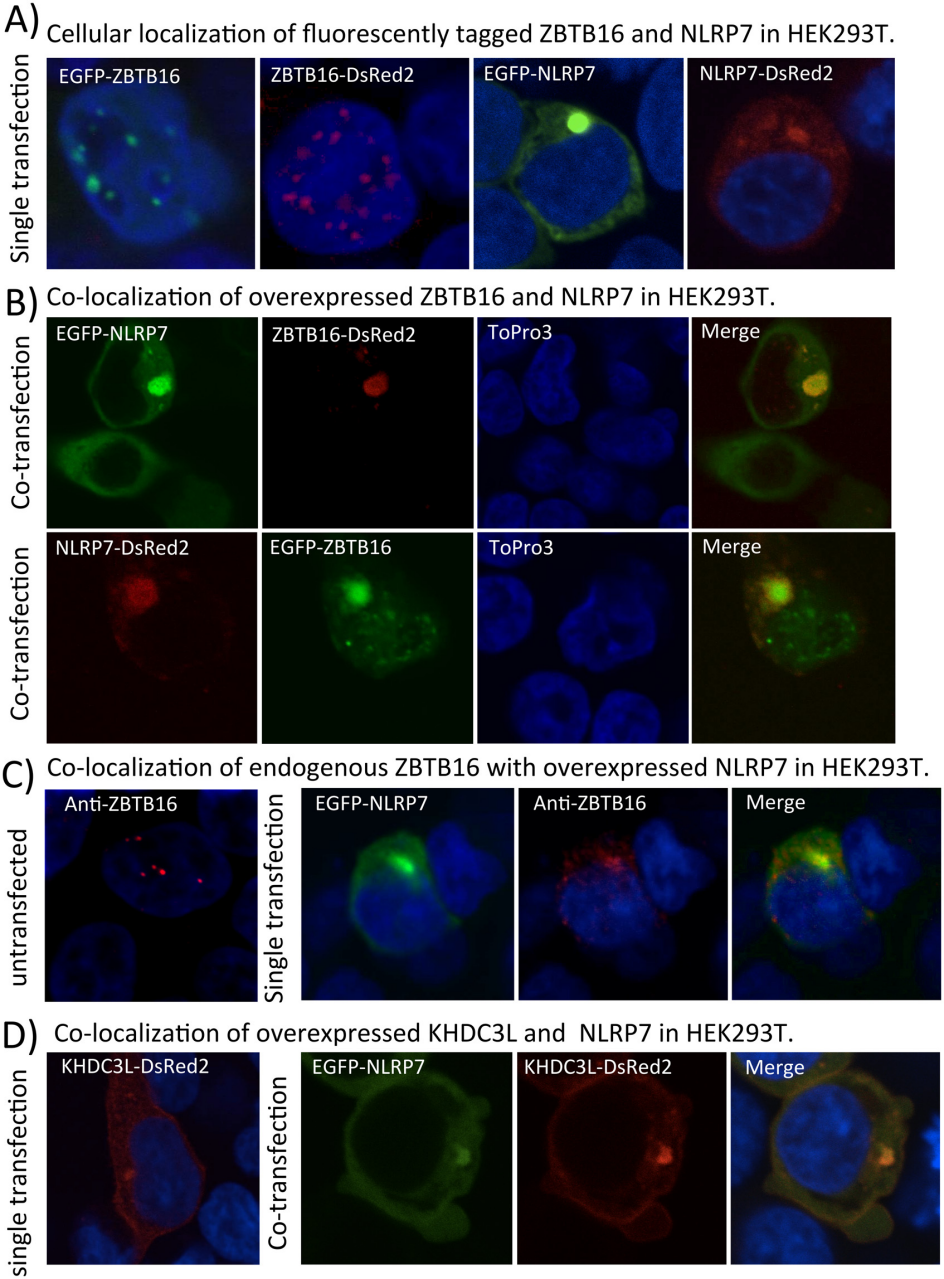
Confocal analysis of ZBTB16 with NLRP7. **A.** Cellular localization of transiently transfected EGFP- (N-terminal) and DsRed2- (C-terminal) tagged NLRP7 or ZBTB16 in HEK293T cells, respectively. Nucleus was counterstained with ToPro3. ZBTB16 localizes in the nuclear speckles, while NLRP7 is distributed in the cytoplasm with occasional accumulation near the nucleus. **B. Upper row:** Co-localization of transiently transfected N-terminal EGFP-tagged NLRP7 and C-terminal DsRed2-tagged ZBTB16 in HEK293T cells. Both proteins co-localize in the cytoplasm near the nucleus. ZBTB16 is re-localized from the nucleus (stained with ToPro3) to the cytoplasm. **Lower row:** Co-localization of transiently transfected C-terminal DsRed2-tagged NLRP7 and N-terminal EGFP-tagged ZBTB16 in HEK293T. Again, both proteins co-localize in the cytoplasm in a juxtanuclear aggregate while some amounts of EGFP-tagged ZBTB16 remain in the nucleus (stained with ToPro3). **C. Single left panel:** Staining of endogenous ZBTB16 in non-transfected HEK293T cells. The protein shows its typical nuclear localization in the nuclear speckles. **Right row:** Transient transfection of N-terminal EGFP-tagged NLRP7 in HEK293T cells results in nuclear export of endogenous ZBTB16 and shows their co-localization in a cytoplasmic juxtanuclear aggregate. **D. Single left panel:** Transient transfection of C-terminal DsRed2-tagged KHDC3L in HEK293Tcells. The protein shows a diffuse distribution in the cytoplasm with occasional accumulation near the nucleus. **Right row:** Transient transfection of N-terminal EGFP-tagged NLRP7 and C-terminal DsRed2-tagged KHDC3L in HEK293T cells. Both proteins co-localize diffusely in the cytoplasm and in juxtanuclear aggregates.

We previously reported that overexpressed NLRP7 is diffusely localized in the cytoplasm of HEK293T cells with occasional aggregations near the nucleus [4]. Therefore, we wanted to investigate whether ZBTB16 co-localizes with NLRP7 and whether the four HYDM1-linked mutations have any influence on the localization of NLRP7 and ZBTB16. It is already known that the transcription factor ZBTB16 localizes in the nucleus within specialized nuclear compartments called nuclear speckles [23]. Independent of size and localization of the fluorescent proteins used for confocal analysis (C-terminal EGFP, 25kD or N-terminal DsRed2, 75kD), single transfection of either EGFP-ZBTB16 or ZBTB16-DsRed2 revealed the typical spotty nuclear localization of the transcription factor within the cell (Fig 4A). Also, a single transfection of EGFP- or DsRed2-tagged NLRP7 depicts for both constructs the typical cellular localization, showing either cells with only diffuse cytoplasmic distribution (data not shown) or cells with diffuse cytoplasmic distribution as well as juxtanuclear aggregation (Fig 4A).

A co-transfection of both proteins, either EGFP-NLRP7 with ZBTB16-DsRed2 (Fig 4B upper row) or NLRP7-DsRed2 with EGFP-ZBTB16 (Fig 4B lower row), resulted in a redistribution of good proportion of ZBTB16 from the nucleus to the cytoplasm (more pronounced in the case of ZBTB16-DsRed2 construct: Fig 4B upper part) where both proteins co-localized in juxtanuclear aggregates. Confocal analyses of the four HYDM1-causing missense mutations/variant did not affect the cytoplasmic co-localization with ZBTB16 (data not shown).

Since ZBTB16 is endogenously expressed in HEK293T cells we also investigated the cellular distribution of native ZBTB16 in the presence of overexpressed EGFP-NLRP7. Staining of endogenous ZBTB16 in non-transfected HEK293T cells depicted the typical spotty nuclear localization, while additional transfection of EGFP-NLRP7 led to a redistribution of ZBTB16 from the nucleus to the cytoplasm, where both proteins co-localize in juxtanuclear aggregates (Fig 4C).

A study on KHDC3L in HEK293T cells has reported the subcellular localization of the protein near the nucleus and demonstrated a co-localization with NLRP7 at the same position [17]. Our confocal analysis confirmed these results, presenting KHDC3L-DsRed2 in the cytoplasm near the nucleus, either singly transfected or co-transfected with EGFP-NLRP7 (Fig 4D).

### *In silico* modeling of the protein-protein interaction between NLRP7 and ZBTB16

**Fig 5:**
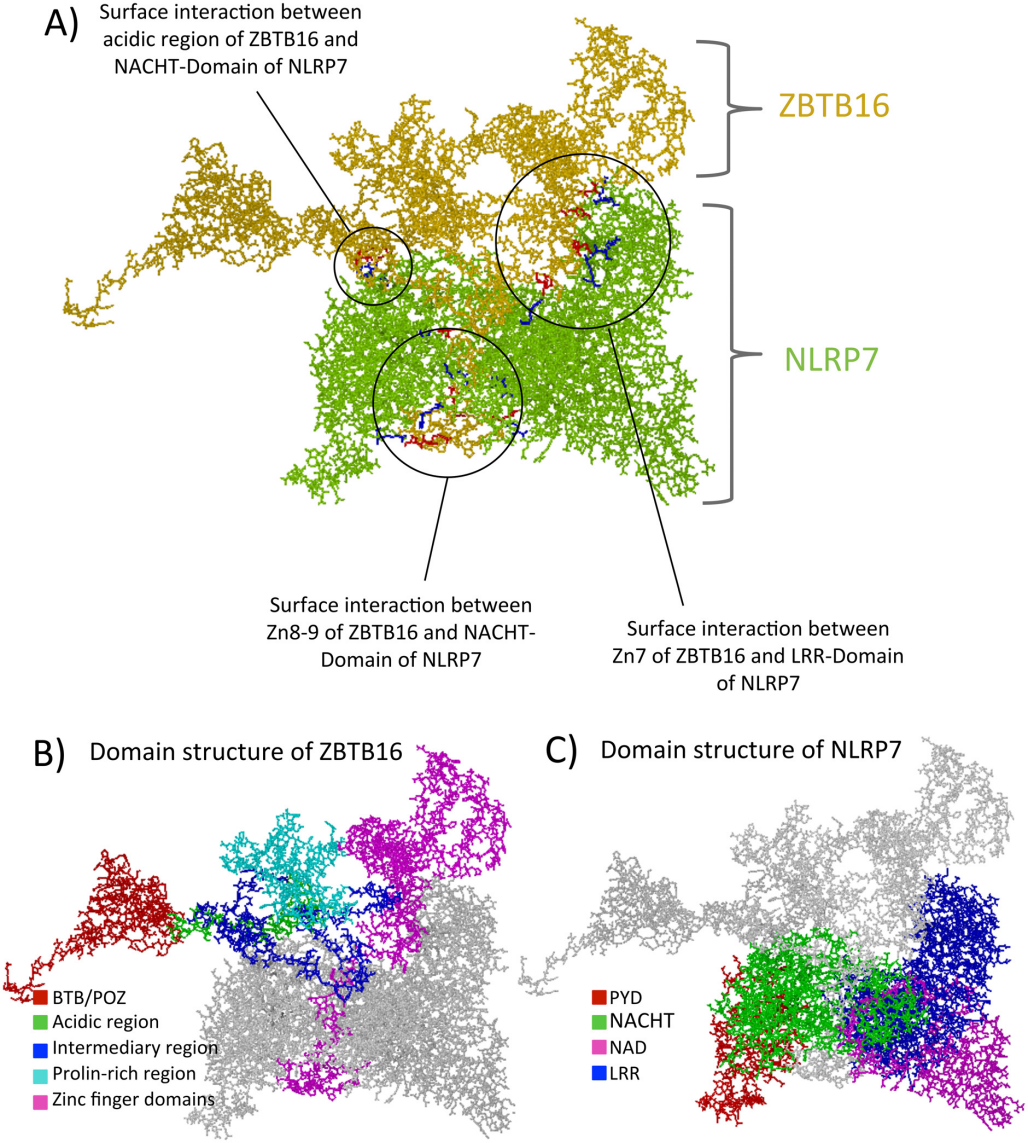
Blind docking of ZBTB16 and NLRP7 threaded protein models. The figure is split into three panels, each representing the ZBTB16/NLRP7 dock in differently colored formats. The models are depicted in stick form. **A.** The first panel shows the proteins ZBTB16 and NLRP7 in yellow and green. The interface regions are marked and specified. The red residues on the interface regions belong to ZBTB16, while the blue ones belong to NLRP7. **B.** and **C**. Both panels show the same docks but with the individual domains of ZBTB16 and NLRP7, respectively, colored according to the code described below. The apposing protein in each panel is uniformly colored in grey.

Since in vitro protein-protein analysis suggested multiple sites of interaction between NLRP7 and ZBTB16, we next attempted to visually verify such multiple interactions. Toward this end, *in silico* structural analysis was performed. Indeed, blind docking of the protein models of ZBTB16 and NLRP7 suggested multiple interacting sites, whereby the following areas of interaction were observed:

**On NLRP7:** Most of the predicted salt bridges and H-bonds occur in the NACHT domain, while some are located at the end of the LRR domain and within the linker region between PYD and NACHT (Fig 5; S1 Table).

**On ZBTB16:** Most of the interaction is observed with the last three zinc finger domains (574–652 amino acid residues) (Fig 5; S1 Table). Some of the interaction is limited to the acidic region of the RD2 domain around amino acid residues 136–139.

The above multiple interactions on both proteins are directly confirming what was observed in the in-vitro cellular work and they reinforce the idea that several domains are needed on both proteins to insure adequate interaction. Interestingly, among the HYDM1-associated mutations investigated in this study, only the residue corresponding to the Leu398Arg missense mutation appears to be close to any of the interaction surfaces (a modeled interaction residue on NLRP7 is the nearby Arg396, S1 Table). In fact, Leu398Arg is the only mutation that induced the interaction in yeast. Thus, it is probably its closeness to the interaction surface that allows this mutation to alter the NLRP7 confirmation and the interaction with ZBTB16 observed in yeast (otherwise not detectable for wild type NLRP7 in yeast).

### DNA methylation analysis after co-transfection of ZBTB16 with wild type or mutant NLRP7

Typical features in HYDM1 are abnormal methylation patterns at DMRs of imprinted genes in the molar tissue. It appeared that the maternal allele was affected. Hence, maternally methylated genes were shown to lose methylation, whereas paternally methylated genes were shown to gain methylation. As ZBTB16 is a transcriptional repressor that mediates its transcriptional silencing through chromatin remodeling via recruitment of specific DNA histone deacetylases and nuclear co-repressors, we next investigated whether both proteins, NLRP7 and ZBTB16, have a co-functional effect on the DNA methylation at imprinted DMRs, using HEK293T cells as adult differentiated somatic cells. However, no significant changes on the methylation levels within paternally (*H19*, *NESP55*) and maternally methylated regions (*PEG3*, *SNRPN*) were observed after a pilot experiment with triplet transient transfections of single NLRP7 (wild type or mutated) or co-transfection with both proteins, NLRP7 and ZBTB16 (S3 and S4 Figs, Experiment 1). As some variations were observed we performed an additional screening of single transiently transfected NLRP7 wild type and both hotspot mutations R693P/R693W, investigating a total number of ten transiently transfected HEK293T cells for each construct. (S3 and S4 Figs, Experiment 2). Again, no significant changes occurred in the analyzed paternally and maternally methylated regions. Therefore, we conclude from overexpressing mutants that NLRP7 is not required to maintain methylation in somatic cells (e.g. HEK293T). Also, this remained unchanged by the presence of ZBTB16. However, a real model using knockouts in relevant cells is still needed.

## Discussion

In this study, we present the transcriptional repressor ZBTB16 as a new interaction partner of NLRP7. A LexA-based Y2H screen identified the ZN-finger domain of ZBTB16 to interact with NLRP7. We verified this interaction in a Gal4 based yeast two-hybrid system. Only the LRR deleted NLRP7 constructs were able to interact with ZBTB16 in the yeast Gal4 system, while no interaction was found between ZBTB16 and full-length NLRP7 (Fig 2A). In contrast, immunoprecipitation analysis in the mammalian cell system led to positive interaction between the full-length proteins (Fig 3A). This discrepancy could be due to the fact that in the yeast system, we are investigating the inactive form of the full-length NLRP7 molecule where the LRR domain is forming a pocket that prevents the NAD domain from freely interacting with ZBTB16 [4]. The involvement of ZBTB16 in a wide range of major developmental and biological processes might provide new links to the molecular mechanism responsible for HYDM1. ZBTB16 mediates its transcriptional silencing through chromatin remodeling via recruitment of DNA histone deacetylases and nuclear co-repressors [24].

### Overexpression of NLRP7 increases the cytoplasmic presence of ZBTB16

Our confocal investigations revealed a clear cytoplasmic juxtanuclear co-localization of NLRP7 and ZBTB16. Moreover, overexpression of NLRP7 increased the cytoplasmic presence of ZBTB16 (Fig 4B and 4C). There are two possible explanations for this observation. First, interference with intracellular cytokine signaling: It was shown that stimulation with cytokine IL-3 redistributes ZBTB16 to the cytoplasm during myelopoiesis [25]. With this re-localization, ZBTB16 decrease its inhibitory function on the chromatin and leads to proliferation and differentiation of human myeloid progenitors. One study has demonstrated that patients with NLRP7 mutations and variants secrete significantly lower amounts of some cytokines, such as IL1B and TNF, in response to LPS [26]. It is therefore possible that wild type and mutated NLRP7 could alter intracellular signaling. Such cytokine-induced re-localization of ZBTB16 to the cytoplasm could mediate the effect of mutated NLRP7 on the DNA, despite its physical absence in the nucleus. Alternatively, physical interaction of NLRP7 that binds to ZBTB16 could trap the latter in the cytoplasm and disrupt its nuclear inhibitory function. This mechanism was previously shown for ZBTB16 in different cell lines, such as fibrosarcoma and keratinocytes, where the soluble form of the metalloprotease heparin-binding EGF-like growth factor H-BEGF-C binds to the zinc-finger domain of ZBTB16 and mediates its nuclear export [27]. However, we cannot provide direct proof for either of the above two scenarios.

Meanwhile, several facts about the biological function of ZBTB16 and the observed pathology of HYDM reinforce this possible involvement. On the one hand, ZBTB16 is highly expressed in stem cells and early progenitor cells, where it maintains their proliferation and maturation [18]. Only a reduced expression of ZBTB16 enables the cells to differentiate into specific cell lineages. On the other hand, a severe HYDM1 phenotype presents an excessive trophoblastic proliferation without the occurrence of differentiated embryonic tissue. Recently, Mahadevan et al. suggested that reduced levels of NLRP7 in hESCs accelerate in vitro differentiation/proliferation into trophoblastic cells [28]. Furthermore, Nguyen et al. suggested that NLRP7 stop mutations lead to an excessive trophoblastic proliferation with a complete absence of embryonic tissues, while less severe mutations still allow the existence of some embryonic differentiated tissues [29]. Therefore, future studies should address how mutations or absence of NLRP7 influence the inhibitory function of ZBTB16 on the chromatin and how this could be linked to a balance between proliferation and differentiation in the relevant cells.

### Are ZBTB16 and NLRP7 putative interaction partners in relevant cells for the manifestation of the disease?

Our methylation analysis did not reveal any functional relationship to the interaction of both proteins in the tested somatic cells (HEK293). It remains unknown in which cells *NLRP7* mutations are relevant for the manifestation of HYDM1. Primordial germ cells are the origin of spermatocytes and oocytes. Before their specification into male or female germ cells they need to epigenetically reprogram their genome to the respective sex of the embryo. A characteristic feature of HYDM1 is the failure to correctly methylate imprinted DMRs on the maternal allele and this implicates that NLRP7 (together with ZBTB16) might play a role in the establishment of the epigenetic identity in the female germline/developing oocyte. The latter could be quite attractive as the nuclear compartment (containing ZBTB16) and may be more open to cytoplasmic proteins (like NLRP7).

Based on our data that show an interaction with the transcription factor (ZBTB16), we suggest that NLRP7 plays a role in the regulatory switch that allows the inner cell mass to differentiate into embryonic tissues. Thus, the interaction of NLRP7 with the transcription machinery is one essential step towards proper embryonic development. However, the exact molecular mechanism remains unknown.

## Supporting Information

S1 Fig NLRP7 yeast two-hybrid screen against an ovarian cDNA library. Ten positive clones matched with the C-terminal segment of human ZBTB16 protein containing the nine zinc finger domains and parts of the RD2 domain. Clones were picked from the primary screening plates, assayed for activity of the second reporter gene lacZ using a quantitative ß-galactosidase assay (HTX assay) and restreaked on new selection plates (Growth on SDhigh). Only the interaction pair that reliably activated both reporter genes (Prey#1) was selected for further analysis.
(PDF)

S2 Fig Immunoprecipitation of NLRP7ΔPYD/LRR. Full-length ZBTB16 and ZBTB16_del4 (Prey-construct from the ovarian library screen) co-precipitated with NLRP7ΔPYD/LRR, while the deletion construct ZBTB16_del5 (containing only the prolin-rich region of RD2 and the first five zinc finger domains) did not co-precipitate with NLRP7ΔPYD/LRR.
(PDF)

S3 Fig Methylation analysis of paternally methylated regions. **Experiment-1:** Methylation of paternally methylated regions H19 and NESP55 after triplet transient transfections of HEK293T with single NLRP7 (wild type or mutated, green dots) or co-transfected with ZBTB16 (red triangle) **Experiment-2:** Methylation of the same paternally methylated regions after a total of ten transient transfections of HEK293T with single NLRP7 (dark green), single R693P (green) and single R693W (light green). Methylation analysis of non-transfected HEK293T cells (blue) and single transfection of an empty vector (brown) served as controls.
(PDF)

S4 Fig Methylation analysis of maternally methylated regions. **Experiment-1:** Methylation of maternally methylated regions PEG3 and SNRPN after triplet transient transfections of HEK293T with single NLRP7 (wild type or mutated, green dots) or co-transfected with ZBTB16 (red triangle) **Experiment-2:** Methylation of the same maternally methylated regions after a total of 10 transient transfections of HEK293T with single NLRP7 (dark green), single R693P (green) and single R693W (light green). Methylation analysis of non-transfected HEK293T cells (blue) and single transfection of an empty vector (brown) served as controls.
(PDF)

S5 Fig Co-immunoprecipitation of ZBTB16 with NLRP7 (complete data). Complete data showing the co-immunoprecipitation of five ZBTB16 deletion constructs del1-5 (Myc-tagged, marked in red) with full-length NLRP7 or one of four NLRP7 deletion constructs (ΔNAD/LRR, ΔLRR, ΔPYD, ΔNACHT; Flag-tagged) using an anti-Flag specific antibody.
(PDF)

S6 Fig Negative and positive controls used for co-immunoprecipitation. Single transfection of all nine different Flag-tagged NLRP7 constructs immunoprecipitated with anti-Flag (upper panel, left side). Single transfection of either Myc-tagged full-length ZBTB16 (upper panel, right) or its five different deletion constructs (lower panel, left) immunoprecipitated with anti-Flag. Co-immunoprecipitation of TAg (Myc-tagged) and p53 (Flag-tagged) served as positive control.
(PDF)

S7 Fig Blue Native PAGE analysis comparing NLRP7 and ZBTB16. Repetition of two more BN-PAGEs representing the second dimension on two different SDS gels: **A.** SDS gel 10% **B.** Gradient SDS gel 4–12%. Monomeric Flag-NLRP7 is visible as a thin line at 113kD, while ZBTB16-Myc occurs as individual spot at its expected size of 75 kD.
(PDF)

S1 Table Interface residues between NLRP7 and ZBTB16. The table shows the putative interaction between the interface residues observed on the ZBTB16/NLRP7 dock. ZN7-9 are the main residues from ZBTB16 interacting either with the NACHT domain or the LRR domain of NLRP7. The interface residue R396, located within the NACHT domain of NLRP7 interacts with the ZBTB16 residue K665 in ZN9 (marked in yellow). R396 is directly located next to L398. The HYDM1 associated missense mutation lead to positive interaction between full-length NLRP7 and ZBTB16 after analysis in the yeast system (see also Fig 2B).
(PDF)

S2 Table List of Primers used in this study. (PDF)
