# Supplementary material for: NLRP7, Involved in Hydatidiform Molar Pregnancy (HYDM1), Interacts with the Transcriptional Repressor ZBTB16
Source: PLoS One. 2015 Jun 29;10(6):e0130416. doi: 10.1371/journal.pone.0130416 (PMC4488268; doi:10.1371/journal.pone.0130416)
Supplement: S1 Fig — Ten positive clones matched with the C-terminal segment of human ZBTB16 protein containing the nine zinc finger domains and parts of the RD2 domain. Clones were picked from the primary screening plates, assayed for activity of the second reporter gene lacZ using a quantitative ß-galactosidase assay (HTX assay) and restreaked on new selection plates (Growth on SDhigh). Only the interaction pair that reliably activated both reporter genes (Prey#1) was selected for further analysis. (PDF) [file pone.0130416.s001.pdf]

Figure S1

Prey Sequence Alignment

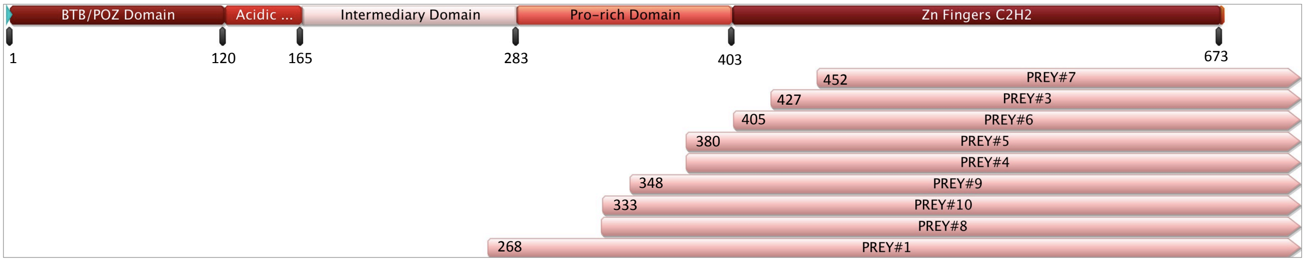

HTX assay results (lacZ)

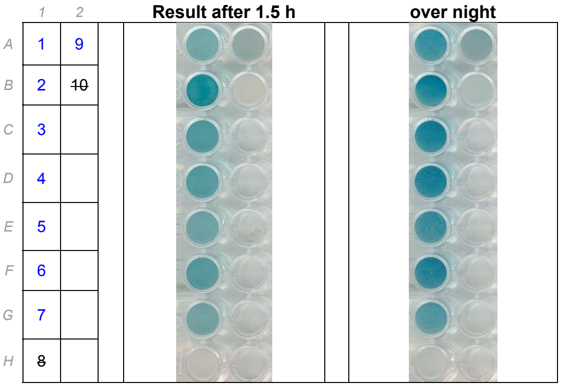

Growth on SDhigh

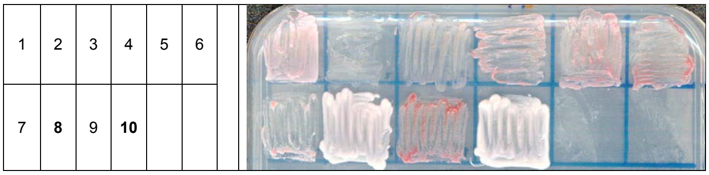

Prey #	Growth on SD-his	lacZ positive	Growth on SD-ade	Interaction
1	+	+++	++	strong
2	+	+++	-	intermediate
3	+	+++	-	intermediate
4	+	+++	+	strong
5	+	+++	+	strong
6	+	+++	+	strong
7	+	+++	-	intermediate
8	+	-	+++	intermediate
9	+	+	+	intermediate
10	+	-	+++	intermediate
