# Supplementary figures and images for: NLRP7, Involved in Hydatidiform Molar Pregnancy (HYDM1), Interacts with the Transcriptional Repressor ZBTB16
Source: PLoS One. 2015 Jun 29;10(6):e0130416. doi: 10.1371/journal.pone.0130416 (PMC4488268; doi:10.1371/journal.pone.0130416)

Figure S2

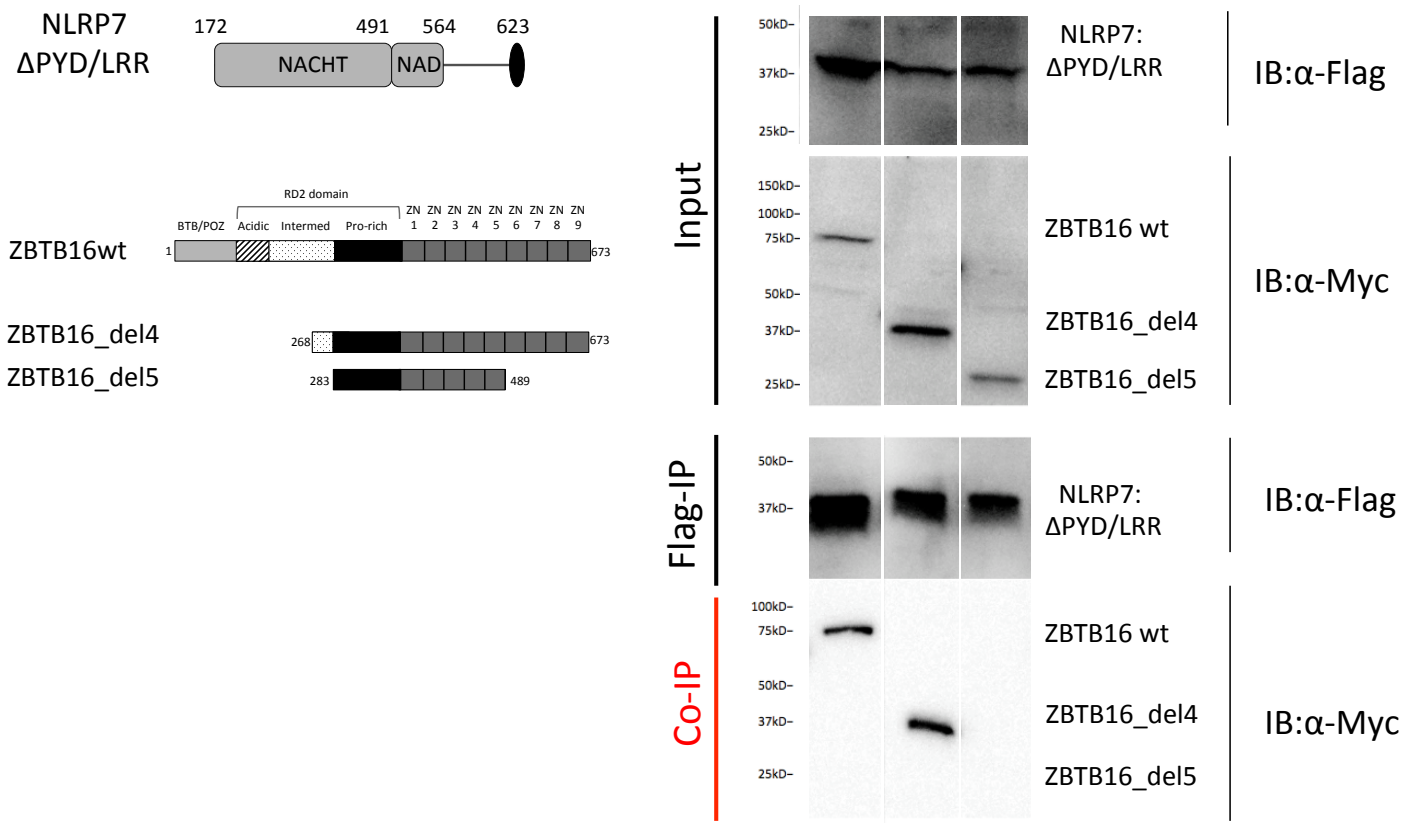

Supplement: S2 Fig — Full-length ZBTB16 and ZBTB16_del4 (Prey-construct from the ovarian library screen) co-precipitated with NLRP7ΔPYD/LRR, while the deletion construct ZBTB16_del5 (containing only the prolin-rich region of RD2 and the first five zinc finger domains) did not co-precipitate with NLRP7ΔPYD/LRR. (PDF) [file pone.0130416.s002.pdf]

Figure S3

# Paternally Methylated

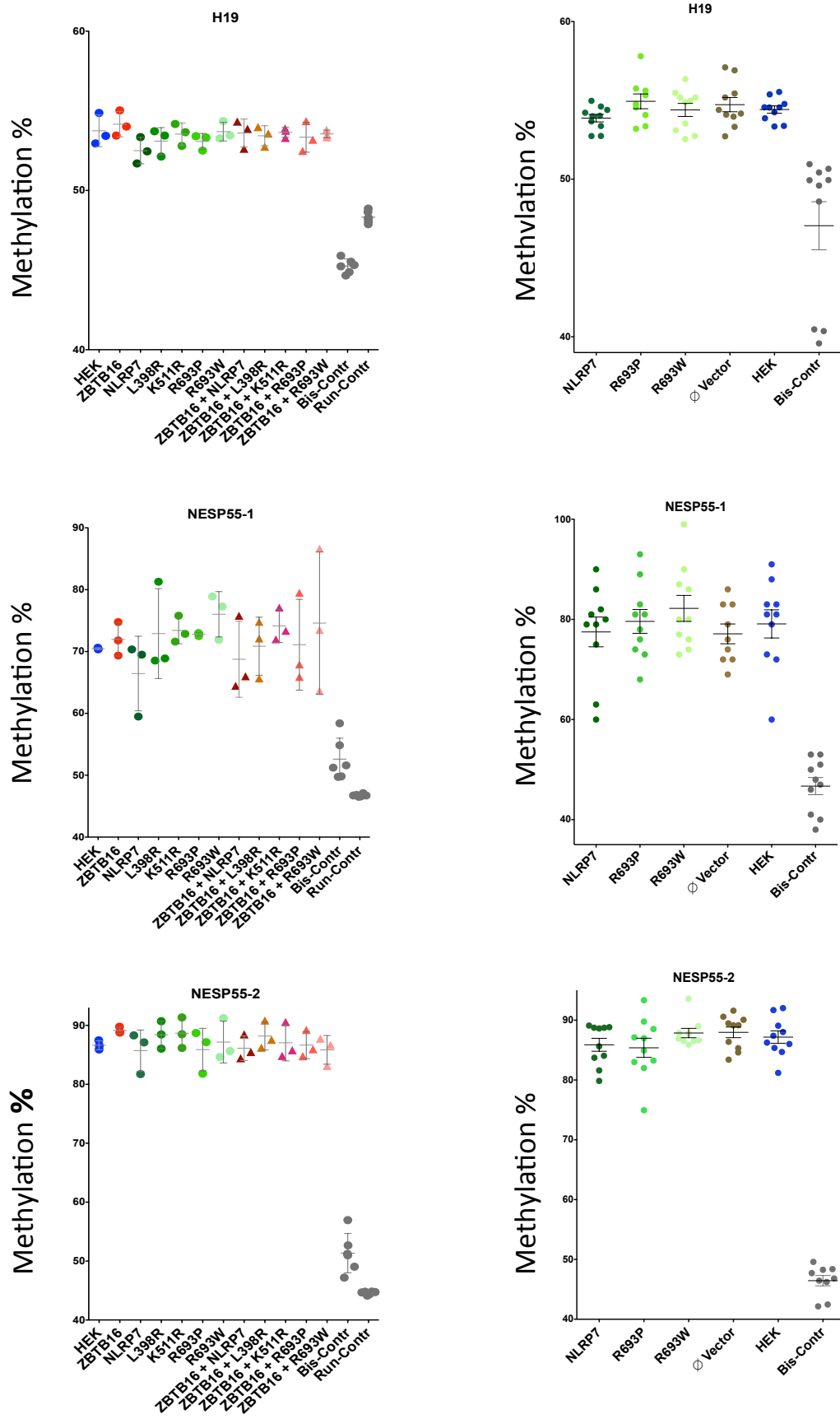

Supplement: S3 Fig — Experiment-1: Methylation of paternally methylated regions H19 and NESP55 after triplet transient transfections of HEK293T with single NLRP7 (wild type or mutated, green dots) or co-transfected with ZBTB16 (red triangle) Experiment-2: Methylation of the same paternally methylated regions after a total of ten transient transfections of HEK293T with single NLRP7 (dark green), single R693P (green) and single R693W (light green). Methylation analysis of non-transfected HEK293T cells (blue) and single transfection of an empty vector (brown) served as controls. (PDF) [file pone.0130416.s003.pdf]

Figure S4

# Maternally Methylated

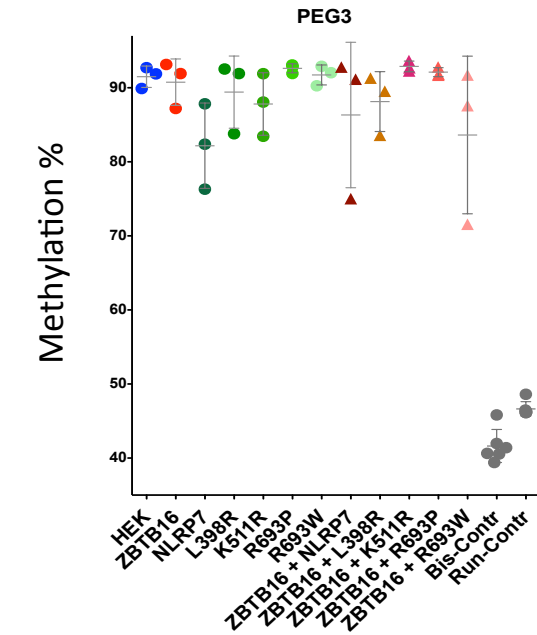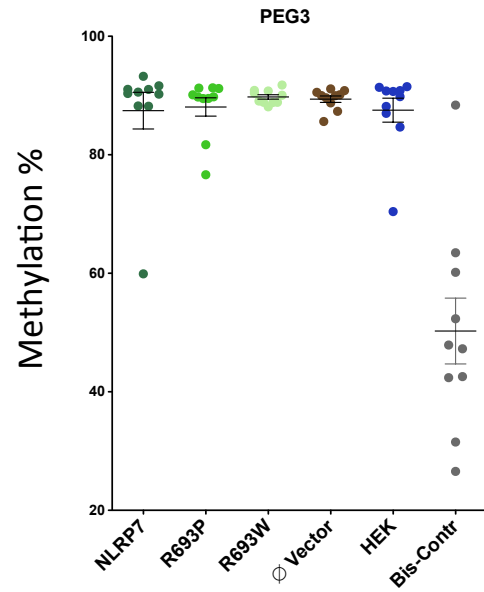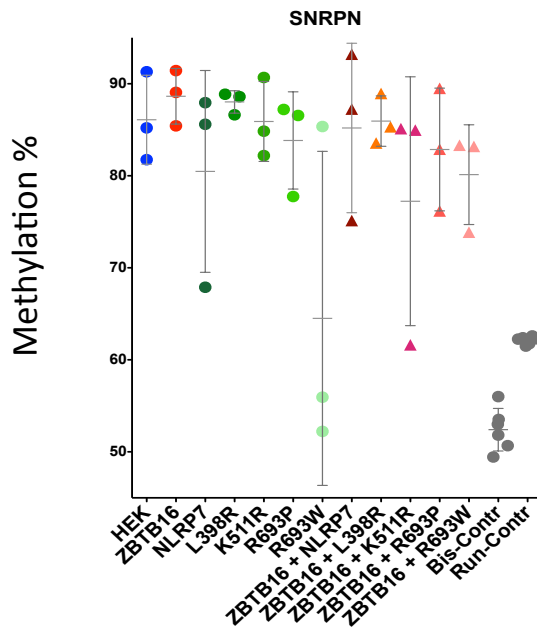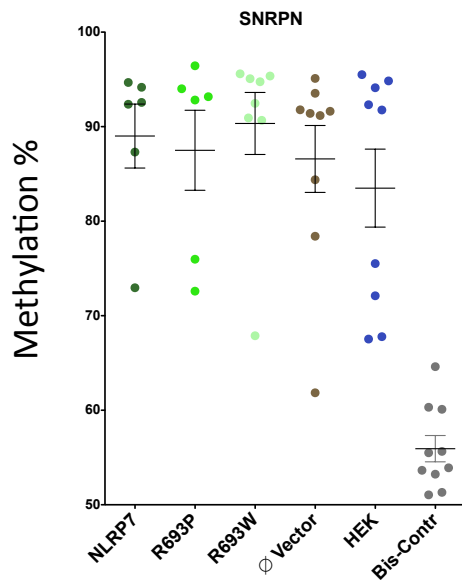

Supplement: S4 Fig — Experiment-1: Methylation of maternally methylated regions PEG3 and SNRPN after triplet transient transfections of HEK293T with single NLRP7 (wild type or mutated, green dots) or co-transfected with ZBTB16 (red triangle) Experiment-2: Methylation of the same maternally methylated regions after a total of 10 transient transfections of HEK293T with single NLRP7 (dark green), single R693P (green) and single R693W (light green). Methylation analysis of non-transfected HEK293T cells (blue) and single transfection of an empty vector (brown) served as controls. (PDF) [file pone.0130416.s004.pdf]

Figure S5

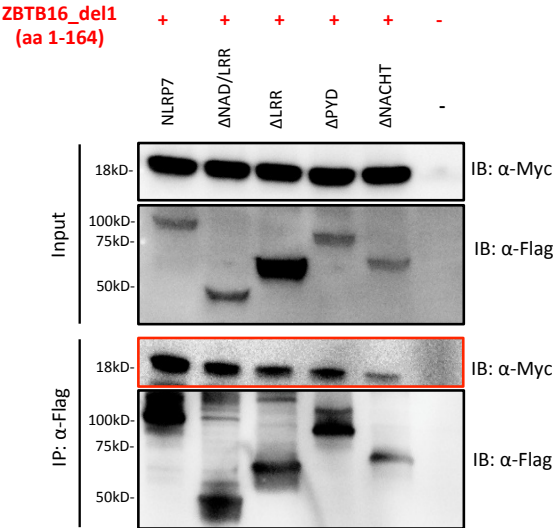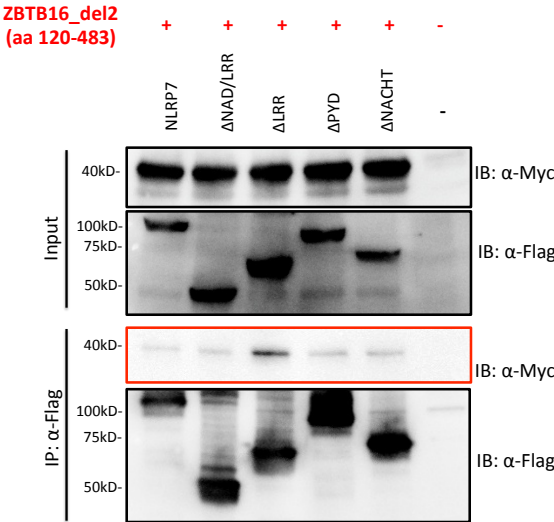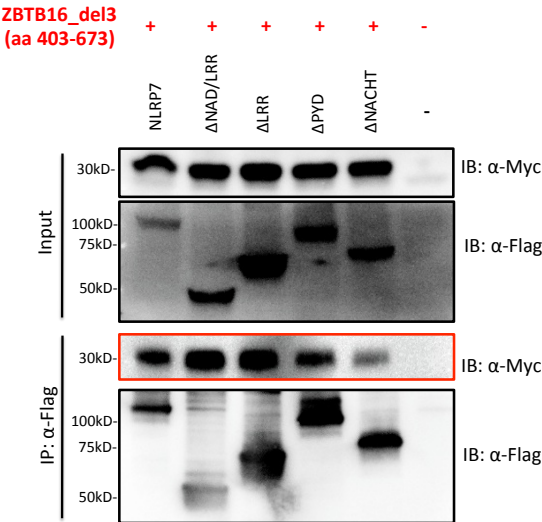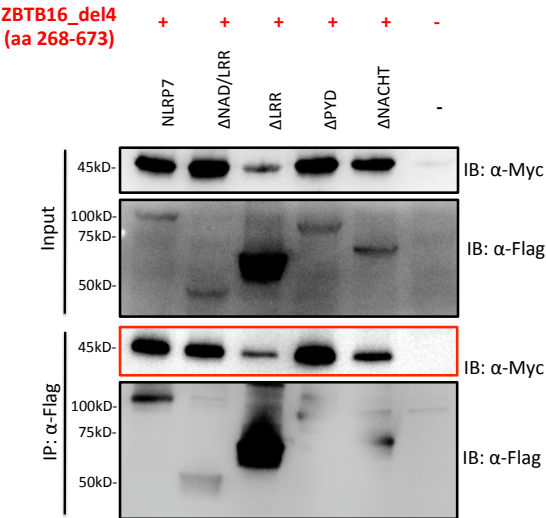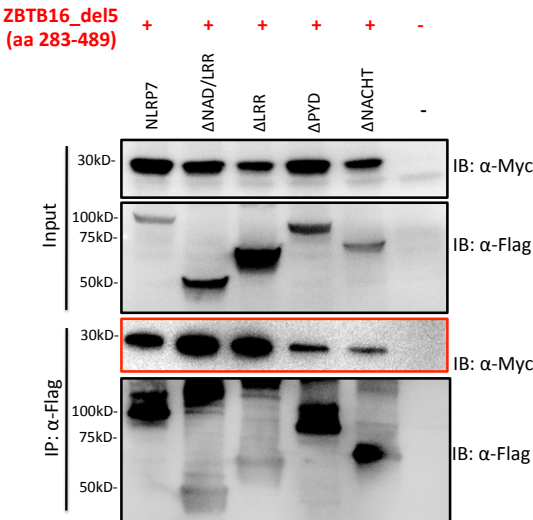

Supplement: S5 Fig — Complete data showing the co-immunoprecipitation of five ZBTB16 deletion constructs del1-5 (Myc-tagged, marked in red) with full-length NLRP7 or one of four NLRP7 deletion constructs (ΔNAD/LRR, ΔLRR, ΔPYD, ΔNACHT; Flag-tagged) using an anti-Flag specific antibody. (PDF) [file pone.0130416.s005.pdf]

Figure S6

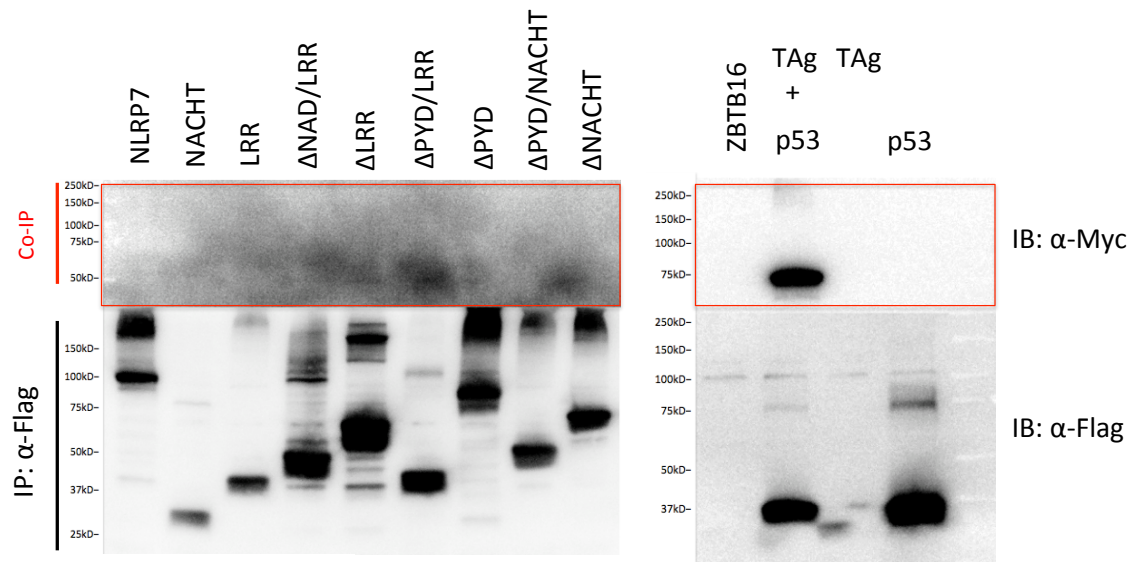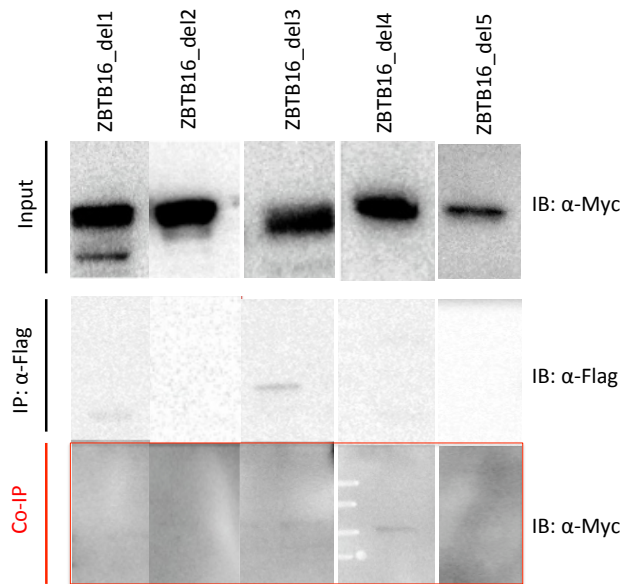

Supplement: S6 Fig — Single transfection of all nine different Flag-tagged NLRP7 constructs immunoprecipitated with anti-Flag (upper panel, left side). Single transfection of either Myc-tagged full-length ZBTB16 (upper panel, right) or its five different deletion constructs (lower panel, left) immunoprecipitated with anti-Flag. Co-immunoprecipitation of TAg (Myc-tagged) and p53 (Flag-tagged) served as positive control. (PDF) [file pone.0130416.s006.pdf]

Figure S7

A

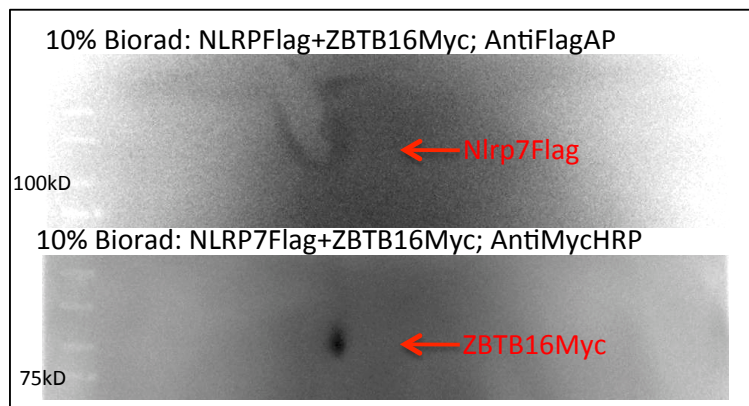

B

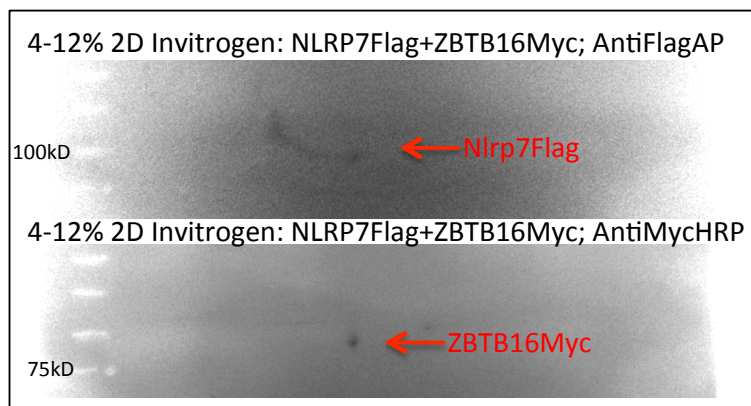

Supplement: S7 Fig — Repetition of two more BN-PAGEs representing the second dimension on two different SDS gels: A. SDS gel 10% B. Gradient SDS gel 4–12%. Monomeric Flag-NLRP7 is visible as a thin line at 113kD, while ZBTB16-Myc occurs as individual spot at its expected size of 75 kD. (PDF) [file pone.0130416.s007.pdf]
