# Supplementary material for: NLRP7, Involved in Hydatidiform Molar Pregnancy (HYDM1), Interacts with the Transcriptional Repressor ZBTB16
Source: PLoS One. 2015 Jun 29;10(6):e0130416. doi: 10.1371/journal.pone.0130416 (PMC4488268; doi:10.1371/journal.pone.0130416)
Supplement: S1 Table — The table shows the putative interaction between the interface residues observed on the ZBTB16/NLRP7 dock. ZN7-9 are the main residues from ZBTB16 interacting either with the NACHT domain or the LRR domain of NLRP7. The interface residue R396, located within the NACHT domain of NLRP7 interacts with the ZBTB16 residue K665 in ZN9 (marked in yellow). R396 is directly located next to L398. The HYDM1 associated missense mutation lead to positive interaction between full-length NLRP7 and ZBTB16 after analysis in the yeast system (see also Fig 2B). (PDF) [file pone.0130416.s008.pdf]

Table S1

Hydrogen bonds

	NLRP7	Position	Dist. [Å]	ZBTB16	Position
1	A:ARG 167[NH2]	PYD-Linker	2.83	B:THR 136[OG1]	Acidic (RD2)
2	A:ARG 167[HE]	PYD-Linker	2.17	B:ASP 139[OD2]	Acidic (RD2)
3	A:ARG 167[NH2]	PYD-Linker	2.80	B:ASP 139[OD2]	Acidic (RD2)
4	A:GLN 155[NE2]	PYD-Linker	3.29	B:ILE 209[O]	Intermed (RD2)
5	A:SER 974[H]	LRR	2.14	B:ASN 577[OD1]	ZN7
6	A:LYS 973[H]	LRR	2.04	B:ASN 577[O]	ZN7
7	A:HIS 970[NE2]	LRR	3.27	B:TYR 593[O]	ZN7
8	A:ALA 916[H]	LRR	2.30	B:GLU 599[OE1]	between ZN7/8
9	A:ARG 917[H]	LRR	2.07	B:GLU 599[OE1]	between ZN7/8
10	A:LYS 889[NZ]	LRR	2.44	B:PRO 601[O]	ZN8
11	A:ARG 455[NH1]	NACHT	3.37	B:LEU 606[O]	ZN8
12	A:ARG 455[NH1]	NACHT	3.28	B:CYS 607[SG]	ZN8
13	A:ARG 455[NH2]	NACHT	3.09	B:CYS 607[SG]	ZN8
14	A:SER 361[H]	NACHT	2.20	B:THR 633[OG1]	ZN9
15	A:ARG 396[NH1]	NACHT	3.25	B:LYS 665[O]	near ZN9
16	A:ARG 396[NH2]	NACHT	3.51	B:LYS 665[O]	near ZN9
17	A:LYS 379[NZ]	NACHT	2.44	B:THR 666[OG1]	near ZN9
18	A:LYS 379[NZ]	NACHT	2.51	B:THR 666[O]	near ZN9
19	A:GLY 219[O]	NACHT	1.99	B:SER 643[H]	ZN9
20	A:GLU 225[OE2]	NACHT	2.73	B:LYS 647[NZ]	ZN9
21	A:ARG 409[O]	NACHT	3.50	B:LYS 653[NZ]	ZN9
22	A:GLU 485[OE1]	NACHT	2.70	B:LYS 653[NZ]	ZN9
23	A:GLN 914[O]	LRR	2.12	B:GLU 599[H]	between ZN7/8

Salt bridges

	NLRP7	Position	Dist. [Å]	ZBTB16	Position
1	A:ARG 167[NE]	PYD-Linker	2.77	B:ASP 139[OD2]	Acidic (RD2)
2	A:ARG 167[NH2]	PYD-Linker	2.80	B:ASP 139[OD2]	Acidic (RD2)
3	A:LYS 889[NZ]	LRR	3.93	B:GLU 599[OE2]	between ZN7/8
4	A:GLU 225[OE1]	NACHT	3.22	B:LYS 647[NZ]	ZN9
5	A:GLU 225[OE2]	NACHT	2.73	B:LYS 647[NZ]	ZN9
6	A:GLU 485[OE1]	NACHT	2.70	B:LYS 653[NZ]	ZN9
