# Supplementary material for: NLRP7, Involved in Hydatidiform Molar Pregnancy (HYDM1), Interacts with the Transcriptional Repressor ZBTB16
Source: PLoS One. 2015 Jun 29;10(6):e0130416. doi: 10.1371/journal.pone.0130416 (PMC4488268; doi:10.1371/journal.pone.0130416)
Supplement: S2 Table — (PDF) [file pone.0130416.s009.pdf]

Table S2: List of Primers used in this study

Yeast-two-Hybrid (Cloning-Primer)		
**Primer**	**Sequenz**	
attB-NLRP7-F	gggg aca agt ttg tac aaa aaa gca ggc ttt-ATG ACA TCG CCC CAG CTA	aa 1-1037
attB-NLRP7-R	gggg ac cac ttt gta caa gaa agc tgg gtt-GCA AAA AAA GTC ACA GCA	
attB-NLRP7-PYD-F	gggg aca agt ttg tac aaa aaa gca ggc ttt-ATG ACA TCG CCC CAG CTA	aa 1-171
attB-NLRP7-PYD-R	gggg ac cac ttt gta caa gaa agc tgg gtt-GCG GCT GAG CTC CTT GCA	
attB-NLRP7-NACHT-F	gggg aca agt ttg tac aaa aaa gca ggc ttt-TAC ACG GTG GTG CTG CAC	aa 172-501
attB-NLRP7-NACHT-R	gggg ac cac ttt gta caa gaa agc tgg gtt-CTG TAC GTC CCC GAT GTC	
attB-NLRP7-NAD-F	gggg aca agt ttg tac aaa aaa gca ggc ttt-AAG CTG CTT TCC GGA GAA	aa 502-623
attB-NLRP7-NAD-R	gggg ac cac ttt gta caa gaa agc tgg gtt-CTG CAG TGA GAG TTT CTG	
attB-NLRP7-LRR-F	gggg aca agt ttg tac aaa aaa gca ggc ttt-GTA GCA AAG GGG GTG TTC	aa 624-1037
attB-NLRP7-LRR-R	gggg ac cac ttt gta caa gaa agc tgg gtt-GCA AAA AAA GTC ACA GCA	
attB-NLRP7-ΔNAD/LRR-F	gggg aca agt ttg tac aaa aaa gca ggc ttt-ATG ACA TCG CCC CAG CTA	aa 1-501
attB-NLRP7-ΔNAD/LRR-R	gggg ac cac ttt gta caa gaa agc tgg gtt-CTG TAC GTC CCC GAT GTC	
attB-NLRP7-ΔLRR-F	gggg aca agt ttg tac aaa aaa gca ggc ttt-ATG ACA TCG CCC CAG CTA	aa 1-623
attB-NLRP7-ΔLRR-R	gggg ac cac ttt gta caa gaa agc tgg gtt-CTG CAG TGA GAG TTT CTG	
attB-NLRP7-ΔPYD/LRR-F	gggg aca agt ttg tac aaa aaa gca ggc ttt-TAC ACG GTG GTG CTG CAC	aa 172-623
attB-NLRP7-ΔPYD/LRR-R	gggg ac cac ttt gta caa gaa agc tgg gtt-CTG CAG TGA GAG TTT CTG	
attB-NLRP7-ΔPYD-F	gggg aca agt ttg tac aaa aaa gca ggc ttt-TAC ACG GTG GTG CTG CAC	aa 172-1037
attB-NLRP7-ΔPYD-R	gggg ac cac ttt gta caa gaa agc tgg gtt-GCA AAA AAA GTC ACA GCA	
attB-NLRP7-ΔNACHT-F	gggg aca agt ttg tac aaa aaa gca ggc ttt-ATG ACA TCG CCC CAG CTA	aa 1-171_492-1037
attB-NLRP7-ΔNACHT-R	gggg ac cac ttt gta caa gaa agc tgg gtt-GCA AAA AAA GTC ACA GCA	
attB-ZBTB16-F	gggg aca agt ttg tac aaa aaa gca ggc ttt- ATG GAT CTG ACA AAA ATG	aa 1-673
attB-ZBTB16-R	gggg ac cac ttt gta caa gaa agc tgg gtt- CAC ATA GCA CAG GTA GAG	
attB-KHDC3L-F	gggg aca agt ttg tac aaa aaa gca ggc ttt- ATG GAC GCT CCC AGG CGG	aa 1-217
attB-KHDC3L-R	gggg ac cac ttt gta caa gaa agc tgg gtt- TAA TCT AGT AAC TGG GTC	

Co-Immunoprecipitation (Cloning-Primer)		
**Primer**	**Sequenz**	
Flag-NLRP7-F	gac tac aaa gac gac gat gac aag ggt acc ATG ACA TCG CCC CAG CTA GA	aa 1-1037
Flag-NLRP7-R	gcg ggt tta aac ggg ccc tct aga ctc gag **TCA** GCA AAA AAA GTC ACA GC	
Flag-NLRP7-NACHT-F	gac tac aaa gac gac gat gac aag ggt acc-TAC ACG GTG GTG CTG CAC GG	aa 1-171
Flag-NLRP7-NACHT-R	gcg ggt tta aac ggg ccc tct aga ctc gag- **TCA** CTG TAC GTC CCC GAT GT	
Flag-NLRP7-LRR-F	gac tac aaa gac gac gat gac aag ggt acc-GTA GCA AAG GGG GTG TTC CT	aa 624-1037
Flag-NLRP7-LRR-R	gcg ggt tta aac ggg ccc tct aga ctc gag- **TCA** GCA AAA AAA GTC ACA GC	
Flag-NLRP7-ΔNAD/LRR-F	gac tac aaa gac gac gat gac aag ggt acc-ATG ACA TCG CCC CAG CTA GA	aa 1-501
Flag-NLRP7-ΔNAD/LRR-R	gcg ggt tta aac ggg ccc tct aga ctc gag- **TCA** CTG TAC GTC CCC GAT GT	
Flag-NLRP7-ΔLRR-F	gac tac aaa gac gac gat gac aag ggt acc-ATG ACA TCG CCC CAG CTA GA	aa 1-623
Flag-NLRP7-ΔLRR-R	gcg ggt tta aac ggg ccc tct aga ctc gag- **TCA** CTG CAG TGA GAG TTT CT	
Flag-NLRP7-ΔPYD/LRR-F	gac tac aaa gac gac gat gac aag ggt acc-TAC ACG GTG GTG CTG CAC GG	aa 172-623
Flag-NLRP7-ΔPYD/LRR-R	gcg ggt tta aac ggg ccc tct aga ctc gag- **TCA** CTG CAG TGA GAG TTT CT	
Flag-NLRP7-ΔPYD-F	gac tac aaa gac gac gat gac aag ggt acc-TAC ACG GTG GTG CTG CAC GG	aa 172-1037
Flag-NLRP7-ΔPYD-R	gcg ggt tta aac ggg ccc tct aga ctc gag- **TCA** GCA AAA AAA GTC ACA GC	
Flag-NLRP7-ΔNACHT-F	gac tac aaa gac gac gat gac aag ggt acc-ATG ACA TCG CCC CAG CTA GA	aa 1-171_492-1037
Flag-NLRP7-ΔNACHT-R	gcg ggt tta aac ggg ccc tct aga ctc gag- **TCA** GCA AAA AAA GTC ACA GC	
MYC-ZBTB16-F	aag ctt ggt acc gag ctc gga tcc act agt- ATG GAT CTG ACA AAA ATG GG	aa 1-673
MYC-ZBTB16-R	gcc ctc tag act cga gcg gcc gcc act gtg- CAC ATA GCA CAG GTA GAG GT	
MYC-ZBTB16_del1-F	aag ctt ggt acc gag ctc gga tcc act agt- ATG GAT CTG ACA AAA ATG GG	aa 1-164
MYC-ZBTB16_del1-R	gcc ctc tag act cga gcg gcc gcc act gtg- CTC CTC GCT GGA ATG CTT CG	
MYC-ZBTB16_del2-F	aag ctt ggt acc gag ctc gga tcc act agt- **ATG** AAG ATG CTG GAG ACC ATC	aa 120-483
MYC-ZBTB16_del2-R	gcc ctc tag act cga gcg gcc gcc act gtg- ATG GGT CTG CCT GTG TGT CC	
MYC-ZBTB16_del3-F	aag ctt ggt acc gag ctc gga tcc act agt- **ATG** GGA GAG CAG TGC AGC GTG	aa 403-673
MYC-ZBTB16_del3-R	gcc ctc tag act cga gcg gcc gcc act gtg- CAC ATA GCA CAG GTA GAG GT	
MYC-ZBTB16_del4-F	aag ctt ggt acc gag ctc gga tcc act agt- ATG GGG GAC AAG GTT GAG GA	aa 268-673
MYC-ZBTB16_del4-R	gcc ctc tag act cga gcg gcc gcc act gtg- CAC ATA GCA CAG GTA GAG GT	
MYC-ZBTB16_del5-F	aag ctt ggt acc gag ctc gga tcc act agt- **ATG** CCG ACT CGA AGC AGC GT	aa 283-489
MYC-ZBTB16_del5-R	gcc ctc tag act cga gcg gcc gcc act gtg- GGC CAT GTC AGT GCC AGT AT	
MYC-KHDC3L-F	aag ctt ggt acc gag ctc gga tcc act agt- ATG GAC GCT CCC AGG CGG	aa 1-217
MYC-KHDC3L-R	gcc ctc tag act cga gcg gcc gcc act gtg- TAA TCT AGT AAC TGG GTC	

Confocal microscopy (Cloning-Primer)

Primer	Sequenz	
EGFP-NLRP7-F	tac aag tcc gga ctc aga tct cga gct caa_ATG ACA TCG CCC CAG CTA GA	aa 1-1037
EGFP-NLRP7-R	cag tta tct aga tcc ggt gga tcc cgg gcc_**TCA** GCA AAA AAA GTC ACA GC	
DsRed2-NLRP7-F	cta gcg cta ccg gac tca gat ctc gag ctc- ATG ACA TCG CCC CAG CTA GA	aa 1-1037
DsRed2-NLRP7-R	ggc cat ggt ggc gac cgg tgg atc ccg ggc- GCA AAA AAA GTC ACA GCA CG	
DsRed2-ZBTB16-F	cta gcg cta ccg gac tca gat ctc gag ctc- ATG GAT CTG ACA AAA ATG GG	
DsRed2-ZBTB16-R	ggc cat ggt ggc gac cgg tgg atc ccg ggc- CAC ATA GCA CAG GTA GAG G	aa 1-673
DsRed2-KHDC3L-F	cta gcg cta ccg gac tca gat ctc gag ctc- ATG GAC GCT CCC AGG CGG	aa 1-217
DsRed2-KHDC3L-R	ggc cat ggt ggc gac cgg tgg atc ccg ggc- TAA TCT AGT AAC TGG GTC	

Site-directed mutagenesis

Primer	Sequenz
NLRP7-L398R-F	ctg cgt ttc c**g**c tgc agc cgg ttc ccg cag
NLRP7-L398R-R	ccg gct gca g**c**g gaa acg cag gaa cag ccc
NLRP7-K511R-F	gaa aga ctc a**g**g aac ccc gac ctg att caa
NLRP7-K511R-R	gtc ggg gtt c**c**t gag tct ttc ttc tcc gga
NLRP7-R693P-F	tct tct gtg c**c**g att ctt tgt gac cac gta
NLRP7-R693P-R	aca aag aat c**g**g cac aga aga gtc act cag
NLRP7-R693W-F	tct tct gtg **t**gg att ctt tgt gac cac gta
NLRP7-R693W-R	aca aag aat cc**a** cac aga aga gtc act cag

BiHot PCR

Primer	Sequenz	
Bi-H19-F1 (Outer)	TTT TTG GTA GGT ATA GAG TT	
Bi-H19-R1 (Outer)	AAA CCA TAA CAC TAA AAC CC	
		H19 (11p15)
Bi-H19-F2 (Nested)	TGT ATA GTA TAT GGG TAT TTT TGG AGG TTT	
Bi-H19-R2 (Nested)	TCC TAT AAA TAT CCT ATT CCC AAA TAA CC (5´Biotin)	
Bi-NESP55-F1 (Outer)	TTT TTT ATT TTA TAG GGT GTA TTT	
Bi-NESP55-R1 (Outer)	AAA ATA AAA TAC TTA AAC ACC AC	
		NESP55 (20q13.2-3)
Bi-NESP55-F2 (Nested)	TTT TTG TAG AGT TAG AGG GTA GGT	
Bi-NESP55-R2 (Nested)	AAA AAA AAC AAC TCA AAA TCT ACC (5´Biotin)	
Bi-PEG3-F1 (Outer)	GGA GAT TTT GTA GTA GTT TTT TAG ATT T	
Bi-PEG3-R1 (Outer)	CCC TTT TCC AAA CCT AAC TTT AAA A	
		PEG3 (19q13.4)
Bi-PEG3-F2 (Nested)	AAA AGG TAT TAA TTA TTT ATA GTT TGG T	
Bi-PEG3-R2 (Nested)	AAA ACT ACT AAT TAA CTA ACA CAA AAA CC (5´Biotin)	
Bi-SNRPN-F1 (Outer)	TCC AAA ACA AAA AAC TTT AAA ACC CAA ATT C	
Bi-SNRPN-R1 (Outer)	AGG TTT TTT TTT ATT GTA ATA GTG TTG TGG GG	
		SNRPN (15q11.2)
Bi-SNRPN-F2 (Nested)	TCA ATA CTC CAA ATC CTA AAA ACT TAA AAT ATC	
Bi-SNRPN-R2 (Nested)	TGT GGG GTT TTA GGG GTT TAG TAG TTT TTT TTT TTT AGG (5`Biotin)	

SNuPE

Primer	Sequenz
H19-SN1	TGG TTG TAG TTG TGG AAT
NESP55-SN1	GTG TTT AAG AGG ATG GAT
NESP55-SN4	GGT ATT TTT TGA GTT TTT
PEG3-SN1	GTT ATT TTG GTT TAG AGT
SNRPN-SN6	TAA GGT TAG TTG TGT

Pyrosequencing

Pyro-H19 (SN1)	TGG TTG TAG TTG TGG AAT
Pyro-NESP55 (SN1)	GTG TTT AAG AGG ATG GAT
Pyro-PEG3 (SN1)	GTT ATT TTG GTT TAG AGT
Pyro-SNRPN	GGG ATT TTT GTA TTG
